# An Evaluation Protocol for A Stabilisation and Referral Area (SARA): A Novel Short Stay Psychiatry Unit Serving A Remote Region of Australia

**DOI:** 10.31083/AP39448

**Published:** 2025-04-01

**Authors:** David Mitchell, Daniel Bressington

**Affiliations:** ^1^Faculty of Health, Charles Darwin University, Casuarina, NT 0810, Australia; ^2^CDU MENZIES School of Medicine, Casuarina, NT 0810, Australia; ^3^Office of the Chief Psychiatrist, NT Health, Darwin, NT 0800, Australia; ^4^Faculty of Nursing, Chiang Mai University, 50200 Chiang Mai, Thailand

**Keywords:** short stay units, short stay psychiatry, emergency psychiatry, service evaluation, models of care

## Abstract

**Background:**

Stabilisation and Referral Areas (SARA) are a unique model of Short Stay Psychiatry inpatient care. This protocol details the comprehensive evaluation of a new SARA service within the Royal Darwin Hospital located in remote and regional Australia. Located in the Northern Territory (NT) there are just 17 specialised mental health beds per 100,000 compared to the national average of 27 per 100,000. There have been no previous evaluations of SARA services in regional and remote Australian settings, therefore their acceptability and potential effects on consumer outcomes in these unique settings is unknown. This study protocol attempts to address this knowledge gap.

**Study Design:**

A mixed method study with triangulation and including mirror methodology.

**Methods:**

A service evaluation protocol is proposed to be conducted over an initial 12 months period with a mirror image component to enable comparison of consumer outcomes prior to the service inception. The service evaluation is guided by the “Reach, Effectiveness, Adoption, Implementation and Maintenance” (RE-AIM) framework and utilized both qualitative and quantitative measures to comprehensively describe the service.

**Results:**

Results will include both qualitative and quantitative data using the “R”, “E” and “A” component (Reach, Effectiveness and Adoption) of the RE-AIM framework.

**Conclusions:**

Emergency departments (EDs) are not well suited to persons experiencing mental health crisis and efforts need to be made to improve the delivery of service as well as patient flow. Minimizing wait times in ED is paramount. SARA is an innovative model of care that may address some of these issues. Evaluating its performance across a range of measures is key to improving and progressing the service. The unique context of the service location which has a large First Nations population and its remote setting adds further weight to the need to understand this model within this geographical context.

## Main Points

1. Emergency Departments are not ideal locations for persons suffering from psychological distress and concerted efforts should be made to improve patient flow from the Emergency Department to definitive care, including inpatient admission. Short Stay Psychiatry Services are a model of care that may provide some utility in addressing the issue of patient flow within the Emergency Department setting.

2. Stabilisation and Referral Areas (SARA) is a short stay psychiatry model of care that may address some of these patient flow issues.

3. This paper outlines a protocol to comprehensively evaluate a novel SARA service being implemented at the Royal Darwin Hospital is located in the remote and regional area of the Northern Territory, Australia.

4. It is envisioned that findings of the study will provide insights and learnings into the model of care within this unique setting with potential application to other remote settings.

## 1. Introduction

This protocol provides details of the proposed comprehensive evaluation of a new clinical short-term mental health inpatient service at Royal Darwin Hospital (RDH). The Stabilisation, Assessment and Referral Area (SARA) is a six-bedded inpatient unit providing timely short-term care and treatment (up to 72 hours) for mental health patients who may require a period of assessment and monitoring as an alternative to traditional acute inpatient admissions, and an alternative to waiting for a mental health bed in the Emergency Department (ED). The unit will operate 24/7 and comprises nursing staff with daily medical support. Allied Health support is also available in a more limited capacity. The SARA, although located above the Inpatient Unit is connected to the ED and shares a collaborative Model of Care designed to enhance the liaison between ED and Mental Health. The SARA clinical governance is discrete from ED with reporting pathways to the Mental Health Clinical Service Unit. The unit is due to open in the second quarter of 2025.

### 1.1 Background

Globally, mental health is paramount to the wellbeing of the population [1]. In Australia, an estimated in 5 people aged 16–85 will experience a mental disorder [1, 2]. More specifically in the Northern Territory (NT), the mental health burden appears even greater. The NT, the most northern and remote region of Australia, has a population that includes approximately 30 % Aboriginal persons with a higher burden of mental illness and a death by suicide rate that is three times the national average [3]. Social disadvantage as well as remoteness add further complication [3]. The Royal Darwin Hospital, in the Top End Region of the NT, is the major tertiary hospital centre for the region. It has long struggled with bed flow within the emergency department-mental health interface. It is likely that bed availability contributes to this. The NT has just 17 specialised mental health beds per 100,000 compared to the national average of 27 per 100,000 [4]. In consequence, persons awaiting a mental health bed potentially spend days in the ED, awaiting transfer to a specialist mental health bed. EDs, by virtue of their design, are not optimal environments for those experiencing a mental health crisis and are reported to have negative impacts on mental health due to the overstimulating environment, lack of privacy and competing clinical needs [5]. Developing models of care that improve patient flow and reduce time in ED is essential to improved patient care and experience.

Worldwide, Psychiatric Short Stay Units (SSUs) have been part of the redesign of other services to improve patient flow through ED [6, 7, 8, 9]. There is evidence, across a range of countries, that SSUs are effective at reducing ED wait times [10]. A SARA is another model of short stay psychiatry. A SARA offers short-term and timely intervention, limited to 72 hours. By moving patients from ED more expediently, they can reduce the burden on the ED. Within the time limited admission, SARA units aim to stabilise persons in an acute mental health crisis, provide further assessment and consider the person’s ongoing referral and treatment needs.

Given, there has never been the application of a short stay psychiatry service within the NT it was timely that this model of care be assessed at the earliest stage of implementation. This is particularly of interest given the unique population of the NT, which is both regional and remote as well as made up of a large proportion of Aboriginal persons. Finally, the evaluation of the SARA, is likely to have applicability to other services, especially those with similar jurisdictions and challenges associated with low per-capita psychiatric bed availability. It is envisioned that the findings from this study will help develop this model but also the implementation of similar models in other regions.

### 1.2 Aims and Objectives

The overall aim of this study is to conduct a comprehensive service evaluation of the new SARA unit at RDH over a 12-month period. The specific objectives are to evaluate: (1) The experiences of stakeholders (including, consumers, family/informal carers and staff associated with the SARA unit); (2) safety of the service; (3) the demographic and clinical characteristics of patients using the service; (4) the efficacy of the SARA unit to meet Key Performance Indicators (KPIs); (5) impact of the SARA unit on the consumer waiting times in ED (during the years pre and post-SARA opening). KPIs include length of stay in the unit (LOS), proportional rates of bed occupancy, 28-day and 7-day re-admission, and 28-day representation to Emergency (Table 1, Ref. [11, 12]).

**Table 1.** **Domains of Evaluation and Outcome Measures Using RE-AIM**.

Domains of Evaluation	Outcome Actions
*Objective 1-	·Experience of stay in SARA
Consumer/Family/Staff Experience	·Experience of care received
(Adoption)	·The performance of SARA staff
(Acceptability)	·Overall level of satisfaction with care
	-Staff Experience
	-Family/Carer Experience
*Objective 2-	Within SARA
Safety	·Incidents of harm to consumers
(Effectiveness)	·Incidents of consumer self-harm
(Efficacy)	·Incidents of Harm to SARA staff
	Within ED
	·Incidents of Aggressive acts in ED
*Objective 3-	SARA Consumer Demographics and Key Performance Indicators (KPI)**
Consumer Journey and ED interface	·Age
(Reach and Effectiveness)	·Sex
(Acceptability and Efficacy)	·Main diagnosis prompting admission
	·% of mental presentations to ED transferring to SARA
	·Average LOS in SARA**
	·% of community discharges from SARA**
	·% of step-up admissions to APU and other**
	·bed occupancy rates**
	·monthly rotation of beds**
	ED interface with SARA including Key Performance Indicators(KPI) **
	·28 day representation to ED post discharge SARA
	·28 day re-admissions through ED
	·7 day re-admissions through ED
	·Average wait time in ED before moving to definitive care
	·Incidence of breaches of the 8 hr NEAT targets
	We will compare ED flow including 4/8/24 hour NEAT target breaches and time spent in ED pre- and post-SARA annually (primary consideration) and quarterly (where sufficient data exist).
	·Average time in ED before managed (mean SD)
	·Breaches 4/8/24 hr NEAT targets
	(number and overall % of contacts)
*Objective 4-	SARA Consumer Demographics
SARA demographics and Diagnostic Related Groups (DRG)	·Age
(Reach)	·Sex
(Acceptability)	·Main diagnosis prompting admission
	Staff and Carer/Family Demographics
	This can be analysed to establish Diagnostic Related Groups using SARA (e.g., Borderline Personality Structure) and thus better develop services to meet consumer needs.

*The R, E and A components evaluated in objectives 1–4 will indirectly assess feasibility and be examined more directly in a follow-up protocol. **Key Performance Indicators (KPI) are based on those set by the Australian Institute of Health and Wellbeing (AIHW) [11] and the 2013 Psychiatric Assessment and Planning Guidelines (Victorian Department of Health) [12]. SARA, Stabilisation and Referral Area; ED, Emergency Department; SD, Standard Deviation; LOS, Length of Stay; APU, Acute Psychiatric Unit; NEAT, National Emergency Assessment and Triage.

## 2. Methods and Materials

### 2.1 Study Design

The service evaluation will be conducted over 12 months, with a mirror-image component to enable comparisons of consumer outcomes in the year prior and one-year post opening of the unit. The service evaluation design was guided by the “RE-AIM” program evaluation framework [13], and this initial evaluation will focus on the R, E and A aspects of the five following evaluative dimensions:

(1) Reach—(assessed by evaluating the number, demographic and clinical characteristics of patients using the service over the duration of the study).

(2) Effectiveness—(assessed by evaluating the safety of the service; efficacy meeting SARA KPIs; impact on consumer waiting times in ED; consumer satisfaction surveys).

(3) Adoption—the use of SARA by different stakeholder groups within the service context over the duration of the evaluation (assessed by exploring the experiences of stakeholders in individual qualitative interviews to better understand contextual factors related to multi-level adoption).

(4) Implementation—the delivery of SARA as intended (will be assessed in a subsequent longer-term evaluation at 24 months after opening and 60 months). This will allow for review of the service once well-established and lays the foundation for assessment of maintenance.

(5) Maintenance—the sustainability of the programme over time (will be, assessed in a subsequent longer-term evaluation at 24 months after opening and after 60 months).

Based on the RE-AIM program evaluation framework, this service evaluation will therefore adopt a prospective cohort mixed-methods study design [14, 15] with a mirror-image analysis of yearly outcomes pre-and-post opening of the service. The evaluation will utilize patient data, ED service use data, quantitative survey-based questionnaires and qualitative data from individual interviews.

The study is a single-service evaluation. Whilst a multicenter evaluation would be ideal, the NT is a small jurisdiction with a population of only 220,000 people [3]. Hence there are no other short-stay psychiatry services in the region. It is envisioned, that owing to the uniqueness of the region, being remote and servicing a large First Nation population, the single service evaluation will prove important to other similar jurisdictions contemplating similar services.

The study will use a mixed method design integrating qualitative and quantitative data to answer specific questions posed by the evaluation. Hence the study will utilize methodological triangulation, integration, and both qualitative and quantitative methods to address the evaluative dimensions. For example, Effectiveness will be assessed by reviewing quantitative SARA KPI data, the effect on waiting times in ED, and a qualitative satisfaction survey. Through this process, there is methodological triangulation to address each individual dimension (R, E, and A). In reviewing individual dimensions there is then an integration of dimensions, evaluating the overall service. In this way, it conceptually aligns with the Mixed Methods Research Trilogy (MMR) that emphasizes a structured approach to integrating mixed data and methods (methods, methodology and philosophy) [14].

### 2.2 Process

The evaluation of SARA, using the R, E and A components of the RE-AIM framework will seek to assess the following attributes of the new model of care: The R and A component (Reach and Adoption) of the study will address aspects of acceptability. The E component efficacy (Effectiveness). The I and M components (Implementation and Maintenance), are envisioned to be examined in a follow-up protocol that will address aspects of feasibility relating to the day-to-day use of the service and its prolonged use. However, the components of R, E, and A will all indirectly assess feasibility as the components of reach, adoption and effectiveness all have practical implications on service development (See Table 1).

### 2.3 Participants 

Consumers (and their de-identified data) will be eligible to be included in the evaluation if they have been admitted to the SARA unit during the evaluation period (1 July 2025–30 June 2026). People who are suitable for the SARA environment include but are not limited to those:

∙ Who are current mental health clients of the RDH;

∙ Exhibiting disturbed behaviour requiring further assessment;

∙ Having complex psychosocial problems causing distress or disturbance in mood/behaviour;

∙ In a situational crisis;

∙ Having a co-morbid physical illness and mental disorder;

∙ Exhibit drug and alcohol problems and concurrent mental disorder;

∙ Adult between 18 and 65 years with special circumstances for an adolescent of 16 years or older;

∙ Brought to ED by Police;

∙ Medically or physically unwell due to self-poisoning, medication overdose or trauma;

∙ Exhibiting self-harming, high risk behaviour or express suicidal ideation or desire;

∙ Who has complex needs and requires integrated medical and psychiatric assessment;

∙ Frequently attending the ED with repeated unresolved mental health concerns.

In addition to those directly admitted to the SARA, we will seek collateral perspective (and de-identified data) of the patient’s family and carers as well as key staff that interact with the SARA, in both the mental health and ED interface. This includes but is not limited to:

(1) Health staff within the SARA and Mental Health Unit (MHU) (Consultant Psychiatrists, Psychiatry registrars, Mental Health Nurses, Allied health, Peer Support and Aboriginal Health Workers).

(2) Health staff within the ED (ED Consultants, ED registrars, nurses and allied health).

(3) Family directly involved in the care of persons admitted to SARA.

### 2.4 Data Collection Process 

#### 2.4.1 Outcome Measures (Quantitative)

RDH service use data including ED and Mental Health, KPIs, as well as the consumer satisfaction surveys will be reviewed (Table 1).

#### 2.4.2 Individual Interviews (Qualitative)

A semi-structured interview guide including probing, follow-up and exit questions will be utilised within the interviews. The facilitator will also ask additional questions to encourage further discussion and elaboration. An interpreter and or local language speaking facilitator will be used for interviews with First Nations people who would prefer to speak their own languages.

### 2.5 Sample Size

Whilst much of the data is descriptive and does not lend itself to a sample size calculation the comparison of ED processing times pre and post establishment of the SARA does require sample size calculation. In previous protocols [9], we established that in order to detect as little as a 5% difference in ED-wait times at 80% power with an alpha value of 0.05, 1006 ED contacts would be required in the pre-SARA and post-SARA sample. The 5% difference in ED-wait times is set pragmatically to detect a change that is measurable and likely to make a discernible difference to an individual patient’s journey and the overall ED flow. Given there are in excess of 1000 mental health ED contacts at the RDH within a 12-month period this sample is achievable within the 12 months of data collection prior and post SARA operation.

The required number of participants for the individual qualitative interviews will be guided by the concept of data saturation; the point at which gathering more data does not reveal new insights about the studied phenomena [16]. Using purposive sampling we will initially interview nine participants from each of the major stakeholder groups (consumers, carers/family members, Health care Professionals). Nine was chosen as an initial target per group because a recent systematic review of data saturation in research using individual qualitative interviews reported that analysis of 9–17 interviews reach data saturation [17]. After nine interviews have been conducted and initially analysed, we will continue recruiting and conducting concurrent analyses until data saturation is apparent. This will entail conducting sets of two additional interviews until data saturation is determined through the analyses.

### 2.6 Quantitative Data Analysis

Descriptive statistical analyses (including means (standard deviations (SDs)) and proportions) will be used to describe the numbers of consumers using the service, the duration/nature of inpatient service use, consumers’ demographic and clinical characteristics, proportion of KPI’s met, and to summarize the demographics characteristics of stakeholders that participate in individual interviews. Inferential statistical analyses will be used to evaluate differences in the ED processing times between the year before and year after the SARA unit opens; these will consist of either paired sample *t*-tests or Wilcoxon signed-rank tests depending on if data meet assumptions for parametric analyses. If sufficient data are available, we will conduct additional analyses of quarterly mean ED processing times pre-and post-opening of SARA and the processing times for consumer sub-groups that have been previously shown to be associated with worse clinical outcomes (for example, First Nations People, people usually residing in rural/remote settings, diagnosis of a severe mental illness, people with substance misuse).

### 2.7 Qualitative Data Analysis

Inductive content analysis will be used for interview data. The audio-recordings of the interviews will be transcribed and cross-checked for accuracy. If interviews have been conducted in local indigenous languages, they will be translated into English by a translator and back-translated by a second translator to check for accuracy. The transcripts will be independently coded by two researchers, and important latent meanings in the data reflected from non-verbal cues will be identified. Codes will be discussed and agreed between the researchers, combined to form subcategories and clustered into categories and overarching themes [18]. Categories and subcategories describing stakeholders’ experiences of SARA will be identified to describe their experiences of the SARA unit and to highlight areas of the service that work well and areas that could be improved. The researchers will discuss and condense the categories and themes to achieve agreement on the meaning of the data. Investigators will clarify any coding/category discrepancies by referring back to the data/interviewees as needed.

Finally, the quantitative and qualitative data will be converged/triangulated to provide a deeper understanding of the quantitative findings and hence provide a comprehensive evaluation of the process and outcomes of the SARA unit.

### 2.8 Ethical/Cultural Considerations, Consent and Data Protection

Ethical approval to conduct the evaluation will be obtained from the University’s research ethics committee and permission to conduct the evaluation will be obtained from the local mental health service provider prior to conducting the evaluation. All study participants engaging in interviews and/or completing questionnaires will be required to provide their informed consent to take part in the study. Informed consent will not be required to use the anonymized service use data and consumers’ clinical/demographic data.

The SARA is predominantly an adult service and potential study participants, whom are inpatients or family members, as well as health staff, will need to be over the age of 18 years, have capacity to consent and a reasonable understanding of the English language. A participant in the study will be given a participant information handout and will need to sign a consent form. Consent is not binding and the participant has the right to withdraw from the study at any point and would not be penalised in any way by this decision.

Within the NT context, with a large First Nation Population, the sensitivities of engagement with research with Indigenous persons is a key consideration. The local Human Research Ethics Committee (HREC) process is very stringent that this is adhered to. The local service and translational research group offers access to culturally appropriate research partners to support the research. Engagement with First Nations persons and those from culturally and linguistically diverse background will be supported by Interpreter Services as well as Cultural Support Workers. Every effort will be made to be culturally safe and avoid any perception of that participants are coerced into the research including information and consent in traditional language. Cultural preferences and kinship considerations will always be addressed with any participant.

Survey data will be collected via Community Care Information System (CCIS) and held on the TEMHS servers before being anonymised and downloaded into a password protected excel file each month for data input/analyses. Data will be held securely in accordance with the university’s and TEMHS’ data protection policies and deleted after 7 years. Data will only be accessed by authorised members of the research team.

### 2.9 Lived Experience Consultation

A co-design approach has been adopted in the establishment of the SARA as part of a broader reform of the RDHs mental health inpatient precinct redesign. This has included extensive consultation with members of the NT lived experience community with carers and consumers representation. This protocol has been specifically reviewed by individuals with lived experience. Consumers with lived experience will also be involved in the interpretation and dissemination of the evaluation findings.

## 3. Discussion

There is considerable attention on the ED environment and its engagement with those in mental health distress [19]. There is universal experience that health systems are struggling with the mental health demands on their ED settings [10]. Whilst there is an increasing demand on ED services to provide care to those with mental health concerns, this is not an optimal environment. Subjectively they can be perceived as highly sensory and disorientating. This likely detracts from providing a therapeutic setting and recovery-orientated care. Persons may find their mental health deteriorating further within this context. This is challenging given the growing mental health demands on ED. How ED services and their associated hospitals adapt and innovated to meet these growing challenges is key to finding solutions.

Developing a short stay psychiatry service closely associated with or embedded in an ED is one way of meeting demand of bed flow between ED and Mental Health Inpatient Services. Ideally this creates a reservoir where patients can be directed for immediate care that is focussed on their mental health and psychosocial needs. It allows for an environment removed from the potential stresses of the ED setting, where patients can be assessed, treated and considerations of future care addressed. Whilst there is growing evidence that short stay psychiatry services can improve wait times in ED [10], there is little available literature on SARA. There is none to our knowledge within a remote context or within a region that services a large proportion of aboriginal persons. Hence there is a further imperative to evaluate and assess the new model of care within this unique setting.

There is limited literature in regard to how best to evaluate mental health services [20, 21, 22, 23]. One suggested method is through the 8 conceptualised dimensions of quality of service. These are (1) Suitability of Service (2) Accessibility of patients to services (3) Acceptance of services by patients (4) Ability of healthcare professionals to provide service (5) Efficiency of health professionals and providers (6) Continuity of service over time (7) Efficiency of Health service and (8) Safety [22]. Most of, if not all of, these concepts can be encapsulated within RE-AIM framework-Reach, Effectiveness, Adoption, implementation and Maintenance. Within this protocol we have specifically addressed Reach, Effectiveness and Adoption. We are cognisant that implementation and maintenance will be pursued in future studies.

Reach involves an understanding of whom the SARA will be serving. There are multiple aspects of this beyond patient care. The SARA will ideally serve patients, but also their families and have impact on the hospital staff that interact with the SARA. Particularly the mental health service staff and the ED staff. Understanding the demographics of each of these groups is key to understanding the service as well as how it effects these important cohorts. Clinical characteristics such as diagnostic related groups (DRG) of the SARA patients is also key and help us better understand those whom are using the service. It is an important consideration in developing the model of care.

Effectiveness of the SARA is a core aspect of this evaluation. The key marker is the impact of the SARA on ED waiting times. This can be conceptualised on multiple levels. This includes metrics of average wait times in ED but also compliance with the Australian standards of the National Emergency Assessment Target (NEAT) target [24]. The NEAT target aims to have all patients in ED assessed, treated and moved to definitive care within 4 hours. This often difficult to achieve marker within the Australian context, is usually augmented by surrogate markers of 8 hours and 24 hours. All three enhance our understanding of the effect and effectiveness of SARA on ED bed-flow. Beyond this, there is a range of KPIs that enhance our understanding of the SARA’s effectiveness. This includes the 28-day and 7-day re-admission rates as well as the 28-day and 7-day representation rates to ED. If the SARA is effective and comprehensive in their care provision, persons admitted to the SARA will ideally not need to return to ED proximal in time to their discharge.

Finally, how the SARA is accepted is an important quality of the service that warrants exploration. This is encapsulated within Adoptability. In building a better picture of the SARA through quantitative and qualitative data we will endeavour to understand how readily this service is accepted. The patient survey and semi-structured interviews of patients, their families and staff are an important consideration in better understanding this.

Overall, using the RE-AIM approach, we are endeavouring to provide a responsive and accurate description of the SARA within its first few years of operation [13]. Such information, provided in a timely manner, is important to the development of the model. It will provide key learnings and understandings of this new model of care and its effect on a variety of stakeholders and associated services such the ED and specialist mental health inpatient unit. Such information is key to responsive adaption and innovation of a burgeoning model of care and its potential utility in solving issues within the ED-Mental Health interface.

### Protocol Strengths and Limitations

There are also strengths and limitations to this service evaluation protocol. The mixed method of qualitative and quantitative data is a key strength, allowing the study to evaluate the model of care across a number of dimensions. The unique context of the NT with a large Aboriginal population provides the opportunity to evaluate the model of care against cultural appropriateness and adoptability. This also has relevance to other similar jurisdictions. In contrast, the study is limited to one site. Ideally, we would like to engage in a multisite evaluation with the advantages of increased power and the ability to study site variability. The reality is that the NT is both small and poorly resourced, therefore such opportunities are not possible. There is also a myriad of confounding variables that complicate any site evaluation in a complex health system that operates in the real world. These are difficult to predict and account for. The study does not have a direct comparison group, other than its ability to analyse the ED environment before and after, using mirror image methodology. The protocol is a single-site evaluation. The population of the NT is comparatively small and remote, with only 220,000 people [3]. Hence there is no other local site for comparison. Ideally, this would be a multicenter evaluation. This limits the ability to make site comparisons and impacts the representativeness beyond the single setting. It is envisioned that there will be indirect comparisons with other settings through any published literature on short-stay psychiatry evaluations, although these will have limited generalizability.

## 4. Conclusions

Emergency Departments are not well suited to persons experiencing mental health crisis and efforts need to be made to improve ED flow. SARA is an innovative and novel short stay psychiatry model of care that may address some of these flow issues within a regional setting with very low bed availability. With the Royal Darwin Hospital acquiring a SARA in mid-2025. It is imperative that this model is evaluated across a range of parameters that include reach, effectiveness and adoptability. This is particularly of interest given the unique population of the NT which is remote and services a large population of First Nations persons. In articulating this protocol, we have endeavoured to be well placed to learn from, improve and further develop this model of care.

## Availability of Data and Materials

Data sharing is not applicable as no data were generated or analyzed.
